# Updating and Validating the Rheumatic Disease Comorbidity Index to Incorporate ICD-10-CM Diagnostic Codes

**DOI:** 10.1002/acr.25116

**Published:** 2023-04-17

**Authors:** Anthony Dolomisiewicz, Hanifah Ali, Punyasha Roul, Yangyuna Yang, Grant W. Cannon, Brian Sauer, Joshua F. Baker, Ted R. Mikuls, Kaleb Michaud, Bryant R. England

**Affiliations:** 1 Veterans Affairs Nebraska-Western Iowa Health Care System, Omaha, NE; 2 Department of Internal Medicine, Division of Rheumatology & Immunology, University of Nebraska Medical Center, Omaha, NE; 3 Salt Lake City VA & University of Utah, Salt Lake City, UT; 4 Corporal Michael J. Crescenz VA & University of Pennsylvania, Philadelphia, PA; 5 FORWARD, The National Data Bank for Rheumatic Diseases, Wichita, KS

**Keywords:** heumatoid arthritis, comorbidity, multimorbidity, outcomes research

## Abstract

**Objective:**

To update and validate the Rheumatic Disease Comorbidity Index (RDCI) utilizing International Classification of Diseases, Tenth Revision, Clinical Modification (ICD-10-CM) codes.

**Methods:**

We defined ICD-9-CM (n=1,068) and ICD-10-CM (n=1,425) era cohorts (n=862 in both) spanning the ICD-9-CM to ICD-10-CM transition in a multicenter, prospective RA registry. Comorbidities were collected from linked administrative data over two-year assessment periods. An ICD-10-CM code list was generated from cross walks and clinical expertise. ICD-9 and ICD-10 derived RDCI scores were compared using Intraclass Correlation Coefficients (ICC). The predictive ability of the RDCI for functional status and mortality during follow-up was assessed using multivariable regression models and goodness-of-fit statistics (Akaike Information Criterion [AIC], Quasi Information Criterion [QICu]) in both cohorts.

**Results:**

Mean (SD) RDCI scores were 2.93 (1.72) for ICD-9-CM and 2.92 (1.74) for ICD-10-CM cohorts. RDCI scores had substantial agreement in individuals spanning both cohorts (ICC 0.71 [0.68–0.74]). Prevalence of comorbidities was similar between cohorts with absolute differences less than 6%. Higher RDCI scores were associated with a greater risk of death and poorer functional status during follow-up in both cohorts. Similarly, in both cohorts, models including the RDCI score had the lowest QICu (functional status) and AIC (mortality) values indicating better model performance.

**Conclusion:**

The newly proposed ICD-10-CM codes for the RDCI generated comparable RDCI scores to those derived from ICD-9-CM codes and are highly predictive of functional status and mortality. The proposed ICD-10-CM codes for the RDCI can be used in rheumatic disease outcomes research spanning the ICD-10-CM era.

## INTRODUCTION

The Rheumatic Disease Comorbidity Index (RDCI) was designed as a tool to quantify the burden of comorbidities and to account for the association of this burden with long-term health outcomes in patients with rheumatic diseases. The RDCI was initially developed using patient questionnaires to collect 10 comorbid conditions, which were weighted to generate a score from 0–9 (1). The RDCI score has been shown to be predictive of physical function, quality of life, and mortality (1,2). Previously, we validated the RDCI score using administrative claims data and International Classification of Diseases, Ninth Revision, Clinical Modification (ICD-9-CM) codes (2). Using claims data, the RDCI compared favorably to other existing general population comorbidity indices in predicting physical function and mortality in people with rheumatoid arthritis (RA) (2). Additionally, the RDCI is more feasible to obtain and a more versatile index, performing well in both self-reported and administrative data (1,2). Because of its performance, feasibility, and versatility, it has been broadly used in RA studies (3) as well as those in psoriatic arthritis (4,5), spondyloarthritis (5–7), gout (8,9), lupus (10), vasculitis (11,12), and osteoporosis (13).

In the U.S., healthcare systems transitioned from the ICD-9-CM classification system for medical conditions to ICD-10-CM on October 1, 2015. The ICD-10-CM coding system contains a nearly five-fold increase in the number of codes available in order to provide improved specificity in classifying medical conditions (14). The transition to ICD-10-CM has required updating the coding of chronic conditions and comorbidity indices that were developed under the previous classification system to confirm validity with ICD-10-CM codes (15–17). The RDCI, derived from ICD-10-CM diagnostic codes, has not yet been validated. Because different comorbidity indices utilize unique definitions of conditions and diagnostic code sets, the generation of an ICD-10-CM code set specific to the RDCI is needed to allow its use in studies spanning both timeframes. Therefore, the objectives of this study were to translate the ICD-9-CM codes used to generate the RDCI to ICD-10-CM codes and to validate the predictive ability for mortality and functional status of the RDCI when derived from ICD-10-CM codes.

## PATIENTS AND METHODS

### Study design and patient population

**Figure 1.**
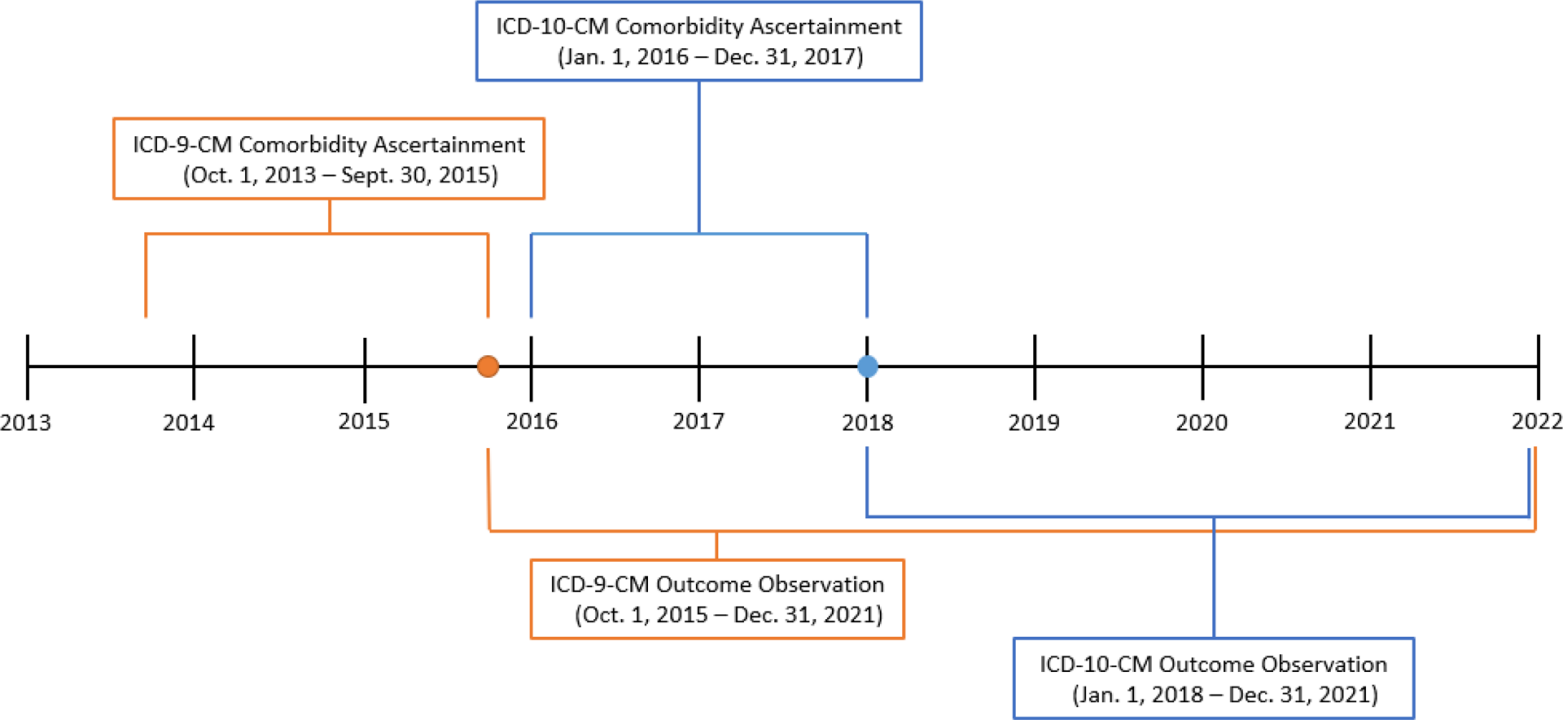
Study design and data collection timelines. ICD-9-CM and ICD-10-CM cohorts were identified over separate time periods immediately before and after the transition to ICD-10-CM. The initial two years were designated as the comorbidity ascertainment period (ICD-9-CM [orange]: October 1, 2013 to September 30, 2015; ICD-10-CM [blue]: January 1, 2016 to December 31, 2017). The outcome observation period started on the index date (colored circles; ICD-9-CM: October 1, 2015; ICD-10-CM: January 1, 2018) and continued until December 31, 2021.

We performed a cohort study within the Veterans Affairs Rheumatoid Arthritis (VARA) registry. This study has been approved by the NWIHCS VA Subcommittee of Human Studies (IRB). The VARA registry is a multicenter prospective cohort of U.S. veterans, >18 years of age who have been diagnosed with RA by a rheumatologist and fulfill the 1987 American College of Rheumatology classification criteria (18). We assembled ICD-9-CM and ICD-10-CM cohorts within VARA over two separate time periods immediately before and after the transition to ICD-10-CM to represent the different classification eras in the U.S. (Figure 1). A subgroup of patients was included in both cohorts. Within each period, the initial two years were designated as comorbidity ascertainment (ICD-9-CM: October 1, 2013 to September 30, 2015; ICD-10-CM: January 1, 2016 to December 31, 2017). Patients were then followed from the index date (ICD-9-CM: October 1, 2015; ICD-10-CM: January 1, 2018) up to December 31, 2021, termed the outcome observation period. A three-month gap between the introduction of ICD-10-CM in the U.S. and the start of our ICD-10-CM cohort was implemented to account for healthcare systems and providers acclimating to the new coding system.

For the current study, VARA participants were included if they were alive as of the index date, had enrolled in the VA at least two years prior to the index date, and had at least one VARA visit during the two years prior to the index date. Participants could contribute to both the ICD-9-CM and ICD-10-CM cohorts.

### Translating ICD-9-CM codes to ICD-10-CM codes

We translated the previously validated ICD-9-CM codes to ICD-10-CM codes using tools available from the websites www.ICD9Data.com and www.ICD10Data.com (2). The websites are owned and operated by Alkaline Software and the tools provide comprehensive lists of ICD-9-CM and ICD-10-CM codes, as well as suggested conversions between the two classification systems (19,20). Utilizing these tools, a list of potential ICD-10-CM codes and their descriptions for each comorbidity in the RDCI was generated (Table 1). The list was reviewed by a physician for clinical relevance and accuracy.

### Comorbidity data collection

We collected comorbid conditions comprising the RDCI by linking the VARA registry participants to national VA administrative databases in the Corporate Data Warehouse (CDW). A condition was considered present if at least one diagnostic code from a VA or non-VA inpatient or outpatient encounter was recorded during the two-year comorbidity ascertainment period. RDCI scores (range 0–9) were calculated from the individual conditions using the original formula: 2 x lung disease + [2 x (heart attack, other CV, OR stroke) OR 1 x hypertension] + fracture + depression + diabetes + cancer + (ulcer or stomach problem) (2).

### Study outcomes for predictive modeling

Mortality and functional status were assessed over the outcome observation period after collection of comorbid conditions. Vital status was determined using linked VA mortality data and functional status was obtained from the VARA registry. Participants in the VARA registry have functional status measured using the Multidimensional Health Assessment Questionnaire (MDHAQ) as part of routine care (21). All MDHAQ values recorded after the index date within the outcome observation period for each cohort were utilized in analyses.

### Study covariates and descriptive variables

Several demographic and RA-related factors were selected as covariates for predictive models or as descriptive variables. Age, sex, race, smoking status (current, former, and never), RA disease duration, and 28-joint disease activity score (DAS28) were obtained from the VARA registry where they are collected by treating providers. These variables were collected at registry enrollment with the exception of the DAS28, which is routinely collected over the course of care. The closest DAS28 value preceding the index date was selected. Anti-cyclic citrullinated peptide (anti-CCP) antibody was measured in a standardized fashion among VARA participants from serum at the time of enrollment, as previously described (22). Use of conventional synthetic disease-modifying anti-rheumatic drugs (csDMARDs), biologic or targeted synthetic DMARDs (b/tsDMARDs), and glucocorticoids as indicated by pharmacy dispensings was obtained from linked VA CDW pharmacy data (23). DMARDs were assessed over the year prior to the index date, while glucocorticoids were assessed over the 90 days prior to the index date.

### Statistical analysis

Patient characteristics were summarized with descriptive statistics. Comorbidities and RDCI scores were descriptively assessed in each cohort separately, as well as among the individuals who were included in both the ICD-9-CM and ICD-10-CM cohorts. Agreement in individual comorbidities among individuals present in both cohorts was assessed using an unweighted Cohen’s Kappa. Intraclass correlation coefficients (ICC) for a two-way mixed effects model with absolute agreement were calculated to assess agreement of the RDCI scores. Kappa and ICC values ranged from 0 to 1 and were interpreted as follows: values less than 0.01 indicating less than chance agreement, values between 0.01 and 0.20 indicating slight agreement, values between 0.21 and 0.40 indicating fair agreement, values between 0.41 and 0.60 indicating moderate agreement, values between 0.61 and 0.80 indicating substantial agreement, and values above 0.81 indicating almost perfect agreement (24–26).

Cox proportional hazards regression models were used to assess the predictive ability of the RDCI score for mortality. We used generalized estimating equations (GEE) models to assess how the RDCI predicted functional status in each coding system. Two sets of covariates were used in regression models. The first model adjusted for age, sex, and race while the fully adjusted model additionally included smoking status, csDMARDs, b/tsDMARDs, glucocorticoids, and anti-CCP positivity.

We subsequently determined the improvement of model performance for both mortality and functional status after including the RDCI score. In mortality analyses, the Akaike’s Information Criterion (AIC) was calculated from Cox regression models with and without the RDCI score. For functional status, QICu values were calculated from GEE models with and without the RDCI. AIC and QICu differences after including the RDCI were calculated to compare model performance, with lower AIC and QICu values indicating better model fit (2). Analyses limiting follow-up in the ICD-9-CM cohort to the maximum follow-up duration of the ICD-10-CM cohort produced similar results as the primary approach (data not shown). Missing covariate data was addressed using the missing covariate indicator method (27). Analyses were conducted using Stata v17 within the VA Informatics and Computing Infrastructure (VINCI) environment.

## RESULTS

### Patient characteristics

Both the ICD-9-CM cohort (n=1,068) and ICD-10-CM cohort (n=1,425) were predominantly male (ICD-9-CM: 89.2%; ICD-10-CM: 87.3%), white (ICD-9-CM: 76.8**%**; ICD-10-CM: 74.0**%**), and had a mean age in the 7^th^ decade (ICD-9-CM: 67.3 ± 10.2 years; ICD-10-CM: 68.2 ± 9.9 years) (Table 2). The majority of patients were anti-CCP antibody positive (ICD-9-CM: 77.5%; ICD-10-CM: 78.1%). The most frequent RA treatments were csDMARDs in both cohorts (ICD-9-CM: 59.5%; ICD-10-CM: 65.3%). There were 862 patients included in both cohorts. The median follow-up time was 6.3 years for the ICD-9-CM and 4.0 years for the ICD-10-CM cohorts.

### Comorbidity prevalence and RDCI scores

RDCI scores were generated using the proposed ICD-9-CM to ICD-10-CM crosswalk codes available in Table 1. The mean ± SD RDCI scores were 2.93 ± 1.72 for the ICD-9-CM cohort and 2.92 ± 1.74 for the ICD-10-CM cohort. Among individuals who were in both cohorts, RDCI scores demonstrated substantial agreement (ICC 0.71 [95% CI 0.68, 0.74]) (Table 3). The prevalence of individual comorbidities in the RDCI was also similar among the cohorts defined during the two coding ascertainment periods, with all absolute differences less than 6% (range: 0.1% to 5.7%). Myocardial infarction, hypertension, diabetes mellitus, depression, stroke, other cardiovascular disease, lung disease, and cancer had moderate agreement or higher (range κ: 0.47 to 0.84) among individuals in both cohorts. Fracture (κ = 0.13) and ulcer/GI problem (κ = 0.27) had slight and fair agreement, respectively.

### Mortality prediction

In the ICD-9-CM cohort, 228 deaths occurred over 5,716 patient-years compared to 210 deaths over 5,144 patient-years in the ICD-10-CM cohort. Higher RDCI scores were associated with a greater risk of death in both cohorts (Table 4; ICD-9-CM: aHR 1.17 [95% CI 1.08, 1.27]; ICD-10-CM: aHR 1.24 [95% CI 1.14, 1.35]). Comparing models without and with the RDCI, model performance improved with the addition of the RDCI in both ICD-9-CM and ICD-10-CM cohorts (Table 4). The reduction in AIC for the ICD-9-CM cohort was 15.29 for the age, sex, and race adjusted model and 11.53 for the fully adjusted model. AIC reductions were even larger in the ICD-10-CM cohort with the reduction in the age, sex, and race adjusted model being 25.93 and the fully adjusted model being 23.85.

### Functional status prediction

The mean ± SD MDHAQ scores during follow-up were 1.0 ± 0.7 in the ICD-9-CM cohort and 0.9 ± 0.6 in the ICD-10-CM cohort. Higher RDCI scores were significantly associated with poorer functional status in both ICD-9-CM and ICD-10-CM cohorts (Table 5; ICD-9-CM: β 0.06 [0.04, 0.08]; ICD-10-CM: β 0.07 [0.05, 0.09]). Functional status prediction was improved in models that included the RDCI in both ICD-9-CM and ICD-10-CM cohorts as indicated by reductions in the QICu (Table 5). The reduction in QICu for the ICD-9-CM cohort was 101.5 in the age, sex and race adjusted model and 89.12 for the fully adjusted model. QICu reduction in the ICD-10-CM cohort was 104.4 in the age, sex, and race adjusted model and 93.2 in the fully adjusted model.

## DISCUSSION

In this study, we aimed to translate the ICD-9-CM codes defined for the RDCI to ICD-10-CM and validate its predictive ability for the key long-term rheumatic disease outcomes of mortality and functional status. We created a set of ICD-10-CM codes that generated comparable RDCI scores and individual comorbidity prevalence estimates to those derived from ICD-9-CM codes within a large, multicenter RA registry, then tested the predictive ability of the RDCI score using the ICD-10-CM code set. We found the ICD-10-CM code set was able to predict mortality and functional status as well as the ICD-9-CM codes. With the updated codes (Table 1), the RDCI can continue to be used in rheumatic disease outcomes research utilizing ICD-10-CM era data.

After assembling an ICD-10-CM code set using a cross walk and clinical expertise, we calculated RDCI scores for RA cohorts spanning ICD-9-CM and ICD-10-CM time periods. RDCI scores were comparable between cohorts (mean scores of 2.93 vs. 2.92) and, among individuals observed during both ICD-9-CM and ICD-10-CM eras, RDCI scores had substantial agreement (K = 0.71). Similarly, when we assessed the prevalence of individual comorbidities between coding systems, the absolute differences were quite small (all <6%). While evaluating individuals in both cohorts, we discovered that agreement between ICD-9-CM and ICD-10-CM varied across individual conditions. Individual conditions with greater chronicity had stronger agreement than acute conditions. For example, diabetes mellitus, hypertension, other cardiovascular disease, lung disease, and depression (chronic conditions) all had at least substantial agreement (K >0.60). In contrast, ulcer or GI problem and fracture of the spine, hip, or leg (acute conditions) had fair and slight agreement (K = 0.27, K = 0.13). We postulate the superior agreement for the more chronic conditions is primarily the result of these conditions being continually addressed and recorded during clinical encounters. However, it is also possible that medical care for acute conditions may have occurred solely outside the VA system (28). Finally, the variability in agreement may be related to differences in the prevalence of these conditions, with those having the lowest prevalence also having poorer agreement.

Others have similarly developed ICD-10-CM code sets for comorbidity indices developed during the ICD-9-CM era. Quan et al. constructed ICD-10-CM coding algorithms for the Charlson and Elixhauser comorbidities using Canadian administrative data (17). They found the frequency of most comorbidities to be similar between ICD-9-CM and ICD-10-CM coding algorithms and the ICD-10-CM code set to either match or outperform the ICD-9-CM versions in predicting in-hospital mortality. As in our study, peptic ulcer disease was a condition that differed in frequency between ICD-9-CM and ICD-10-CM. Glasheen et al. have since updated the ICD-10-CM coding for the Charlson Comorbidity Index, validating the predictive performance of ICD-10-CM codes for hospital admission and mortality (16). Sears and Rundell developed updated ICD-9-CM and ICD-10-CM code lists for the Functional Comorbidity Index (FCI), assessing concordance before and after the transition to ICD-10-CM (15). The frequency of individual comorbidities was consistent between coding algorithms for thirteen of eighteen comorbidities. While the Functional Comorbidity Index retained predictive value for hospital length of stay with ICD-10-CM codes, there was an interaction between the index and coding algorithm suggesting modest reduced performance with ICD-10-CM codes. Our findings are in agreement with these studies; appropriate ICD-10-CM code sets for comorbidity indices can have concordance with ICD-9-CM versions and retain predictive value for health outcomes.

Comorbidity burden is a crucial determinant of long-term outcomes that include mortality and functional status among people with rheumatic diseases (3,22,29–31). Thus, we tested the validity of the ICD-10-CM codes by assessing the predictive ability of the RDCI score for mortality and functional status. As expected, higher RDCI scores, indicating a greater comorbidity burden, were associated with higher mortality risk and poorer functional status during follow-up in both the ICD-9-CM and ICD-10-CM cohorts. Moreover, metrics of overall model performance (AIC, QICu) for predicting these same outcomes in each cohort also showed substantial improvement when the RDCI was included. Together, these findings demonstrate that the RDCI calculated with the proposed ICD-10-CM codes maintains expected predictive value for key long-term outcomes.

There are limitations to this study. The cohorts were predominantly male, white, and of middle to older age which may limit generalizability since RA is a female predominant condition. However, the RDCI was previously found to perform similarly for predicting mortality and functional status in VA and non-VA cohorts (2). The ICD-9-CM cohort was defined during the end of the ICD-9-CM era when clinicians may be more experienced with diagnostic code selection, compared with the ICD-10-CM cohort which was during the early period of the ICD-10-CM era. To reduce misclassification resulting from this, we excluded the initial three-month time period after ICD-10-CM implementation. Patients may have received care outside the VA, which could underestimate the prevalence of individual conditions and RDCI scores, though we do not expect this to differ by ICD era. Finally, we evaluated the predictive ability for mortality and functional status but recognize comorbidity burden is also associated with other long-term outcomes such as quality of life, which were not available.

In conclusion, we generated a set of ICD-10-CM codes for the RDCI that produce comparable RDCI scores and chronic disease prevalence estimates to those derived from previously validated ICD-9-CM codes. RDCI scores calculated using these ICD-10-CM codes are highly predictive of functional status and mortality, comparing favorably to scores based on ICD-9-CM codes. The proposed RDCI codes can be used in rheumatic disease outcomes research spanning the ICD-10-CM era.

## Figures and Tables

**Table 1.** RDCI ICD-9-CM to ICD-10-CM crosswalk

Comorbidity/Condition	ICD-9-CM	ICD-10-CM
Myocardial Infarction	410.x-412.x	I20.0; I21.x-I22.x; I24.x; I25.110; 125.2; I25.700; I25.710; I25.720; I25.730; I25.750; I25.760; I25.790
Hypertension	401.x; 405.x	I10.x; I15.x; I16.x
Diabetes Mellitus	249.x-250.x	E08.x-E11.x; E13.x
Depression	296.2–296.39; 300.4; 311.x	F32.x (excluding F32.81); F33.x; F34.1
Ulcer or stomach problem	531.x–535.7x; 536.3; 536.8–536.9; 578.9	K25.x-K30.x; K31.84; K31.89; K31.9; K52.81; K92.2
Stroke	430.x–431.x; 433.x–435.x; 997.02	G45.x (excluding G45.3 and G45.4); G46.0-G46.4; I60.x-I61.x; I63.x; I65.x-I66.x; I97.81x-I97.82x
Fracture spine, hip, or leg	733.13–733.16; 733.93; 733.96–733.98; 805.x–806.x; 808.x; 820.x–821.x; 823.x; 827.x	M48.4x-M48.5x; M80.05x-M80.06x; M80.08x; M80.85x-M80.86x; M80.88x; M84.35x-M84.36x; M84.45x-M84.46x; M84.55x-M84.56x; M84.65x-M84.66x; M84.75x; S12.x (excluding S12.8x); S22.0x; S32.x; S72.x; S82.1x-S82.2x; S82.311x; S82.312x; S82.319x S82.4x; S82.81x-S82.83x; S82.86x; S82.89x; S82.9x
Other Cardiovascular	394.x–396.x; 402.x; 404.x; 413.x–414.x; 424.0–424.3; 425.x–428.x	I05.x-I06.x; I08.x; I11.x; I13.x; I20.1; I20.8; 120.9; I25.10; I25.111; I25.118; I25.119; I25.3; I25.41; I25.42; I25.5; I25.6; I25.701; I25.708; I25.709; I25.711; I25.718; I25.719; I25.721; I25.728; I25.729; I25.731; I25.738; I25.739; I25.751; I25.758; I25.759; I25.761; I25.768; I25.769; I25.791; I25.798; I25.799; I25.810; I25.811; I25.812; I25.82; I25.83; I25.84; I25.89; I25.9; I34.x-I35.x; I36.x; I37.x; I42.x-I45.x; I46.x; I47.x-I50.x; R00.1
Lung Disease	490.x–493.99; 494.x; 495.x; 496.x; 500.x–505.x; 515.x–517.x; 714.81	J40.x-J47.x; J60.x-J67.x; J84.x; J99; M05.10-M05.19
Cancer	140.x–209.39	C00.x-C26.x; C30.x-C41.x; C43.x-C58.x; C60.x-C75.x; C76.x-C86.x; C88.2-C88.9; C90.x-C93.x; C94.0x-C94.3x; C94.8x; C95.x-C96.x; C7A.x; C4A.x; D03.x

RDCI = Rheumatic Disease Comorbidity Index; ICD-9-CM = International Classification of Diseases, Ninth Revision, Clinical Modification; ICD-10-CM = International Classification of Diseases, Tenth Revision, Clinical Modification

**Table 2.** Patient characteristics by study cohort.

Characteristics	ICD-9-CM Cohort (N = 1,068)	ICD-10-CM Cohort (N = 1,425)
Age, years	67.3 ± 10.2	68.2 ± 9.9
Male sex, no. (%)	953 (89.2)	1,244 (87.3)
White, no. (%)	820 (76.8)	1,054 (74.0)
Smoking status, no. (%)		
Current	264 (24.7)	341 (23.9)
Former	553 (51.8)	740 (51.9)
Never	231 (21.6)	293 (20.6)
RA disease duration, years	15.4 ± 10.9	16.6 ± 11.3
Anti-CCP antibody positive (%)	753 (77.5)	1,051 (78.1)
DAS28-CRP	3.1 ± 1.2	2.9 ± 1.2
RA treatments, no. (%)		
csDMARDs	635 (59.5)	931 (65.3)
b/tsDMARDs	297 (27.8)	470 (33.0)
Glucocorticoids	292 (27.3)	330 (23.2)

Values are reported as mean ± standard deviation unless stated otherwise. Percentages are among those with non-missing values.

Missing data for ICD-9-CM cohort: Smoking status (n=20), Anti-CCP antibody (n=97), and DAS28-CRP (n=269). Missing data for ICD-10-CM: Smoking status (n=51), Anti-CCP antibody (n=79), and DAS28-CRP (n=384).

Abbreviations: ICD-9-CM = International Classification of Diseases, Ninth Revision, Clinical Modification; ICD-10-CM = International Classification of Diseases, Tenth Revision, Clinical Modification; DAS28 = 28-joint Disease Activity Score; RA = Rheumatoid Arthritis; csDMARDs = conventional synthetic disease-modifying antirheumatic drug; b/tsDMARDs = biologic or targeted synthetic disease-modifying antirheumatic drug; Anti-CCP = Anti-cyclic citrullinated peptide

**Table 3.** Agreement of RDCI scores and prevalence of comorbidities

	All participants		Participants in both cohorts (N=862)		
	ICD-9-CM (N=1,068)	ICD-10-CM (N=1,425)	ICD-9-CM	ICD-10-CM	ICC (95% CI)
RDCI Score*	2.93 ± 1.72	2.92 ± 1.74	2.89 ± 1.70	3.02 ± 1.76	0.71 (0.68, 0.74)
	All participants		Participants in both cohorts (N=862)		
Comorbid Condition	ICD-9-CM % (N=1,068)	ICD-10-CM % (N=1,425)	ICD-9-CM %	ICD-10-CM %	Cohen’s Kappa
Myocardial infarction	7.0	4.9	6.5	5.6	0.47
Other cardiovascular	34.6	36.5	32.1	37.8	0.63
Stroke	5.1	6.5	4.5	6.3	0.49
Hypertension	65.5	63.8	65.1	63.2	0.71
Diabetes mellitus	29.0	28.3	27.8	31.2	0.84
Lung disease	29.8	32.3	29.4	33.1	0.62
Cancer	20.5	19.9	20.3	21.8	0.58
Ulcer or GI problem	7.8	7.7	6.8	8.0	0.27
Fracture**	1.7	2.7	1.3	2.0	0.13
Depression	27.6	23.6	28.3	25.8	0.61

* Values mean ± SD unless otherwise noted

** Fracture of the spine, hip, or leg

Abbreviations: GI = Gastrointestinal; ICC = Intraclass Correlation Coefficient; ICD-9-CM = International Classification of Diseases, Ninth Revision, Clinical Modification; ICD-10-CM = International Classification of Diseases, Tenth Revision, Clinical Modification; RDCI = Rheumatic Disease Comorbidity Index

**Table 4.** Performance of the RDCI score for predicting mortality

Model	N/N Deaths	aHR for RDCI (95% CI)	P value	Model AIC (No RDCI)	Model AIC (RDCI)	ΔAIC
ICD-9-CM	1,068/228					
Age, sex, & race		1.19 (1.10, 1.29)	<0.001	2,951.75	2,936.46	−15.29
Fully adjusted*		1.17 (1.08, 1.27)	<0.001	2,930.67	2,919.14	−11.53
**ICD-10-CM**	1,425/210					
Age, sex, & race		1.25 (1.15, 1.36)	<0.001	2,894.92	2,868.99	−25.93
Fully adjusted*		1.24 (1.14, 1.35)	<0.001	2,892.93	2,869.08	−23.85

Values are from Cox regression model.

* Model includes age, sex, race, smoking status, conventional synthetic DMARDS, biologic or targeted synthetic DMARDs, glucocorticoids, and anti-CCP antibody.

Abbreviations: AIC = Akaike Information Criterion; aHR = adjusted hazard ratio; CI = Confidence Interval; ICD-9-CM = International Classification of Diseases, Ninth Revision, Clinical Modification; ICD-10-CM = International Classification of Diseases, Tenth Revision, Clinical Modification; RDCI = Rheumatic Disease Comorbidity Index

**Table 5.** Performance of the RDCI for predicting Multidimensional Health Assessment Questionnaire scores

Model	N/N observations	β for RDCI (95% CI)	P value	Model QICu (No RDCI)	Model QICu (RDCI)	ΔQICu
**ICD-9-CM**	931/8,769					
Age, sex, & race		0.06 (0.04, 0.08)	<0.001	3,485.37	3,383.86	−101.51
Fully adjusted*		0.06 (0.04, 0.08)	<0.001	3,435.45	3,346.33	−89.12
**ICD-10-CM**	1,175/6,864					
Age, sex, & race		0.07 (0.05, 0.09)	<0.001	2,567.83	2,463.43	−104.4
Fully adjusted*		0.07 (0.05, 0.09)	<0.001	2,510.82	2,417.62	−93.2

Values are from generalized estimating equations models.

* Model includes age, sex, race, smoking status, conventional synthetic DMARDS, biologic or targeted synthetic DMARDs, glucocorticoids, and anti-CCP antibody.

Abbreviations: CI = Confidence Interval; COEFF = Coefficient; ICD-9-CM = International Classification of Diseases, Ninth Revision, Clinical Modification; ICD-10-CM = International Classification of Diseases, Tenth Revision, Clinical Modification; QIC = Quasi Information Criterion; RDCI = Rheumatic Disease

Comorbidity Index

## Significance & Innovations

- The Rheumatic Disease Comorbidity Index (RDCI) was developed to capture the contribution of comorbidities to mortality, physical disability, and quality of life in patients with rheumatic diseases.
- Previously developed from patient questionnaires and validated using International Classification of Diseases, Ninth Edition, Clinical Modification (ICD-9-CM), the RDCI has not been updated and validated to the ICD-10-CM system.
- Use of a proposed set of ICD-10-CM codes to generate the RDCI produced comparable RDCI scores, comorbidity prevalence, and prediction of mortality and functional status as the ICD-9-CM derived RDCI.
- With the proposed code set, the RDCI can be used in rheumatic disease outcomes research encompassing the ICD-10-CM era.

## Abbreviations:

ICD-9-CM: International Classification of Diseases, Ninth Revision, Clinical Modification
ICD-10-CM: International Classification of Diseases, Tenth Revision, Clinical Modification
